# The American and Southern Dental Society Clinics and Exhibits

**Published:** 1888-12

**Authors:** 


### THE AMERICAN AND SOUTHERN DENTAL SOCIETY CLINICS AND EXHIBITS.

Several things combined to operate against the clinics. In the first place no special time had been set apart beforehand for clinics, and the practical work was thus thrown in the background. Then the weather was so bad that no one had energy enough to take hold and manage them. The separate business meetings of the two associations, and the two joint meetings daily, followed so closely upon one another, that each day was fully occupied, and no opportunity was found for clinics, except a clinic in continuous gum work, by Dr. L. P. Haskell, and one in aluminum casting, by Dr. C. C. Carroll.

The exhibits, on the other hand, were fully up to the standard of former years. A new feature was introduced into the exhibit in the shape of many new electrical appliances. Dr. Starr, of the S. S. White Dental Manufacturing Co., had several novel and useful appliances. The motor power was taken directly from the electric wires and a governor or regulator introduced to control the current and prevent shock. It seems as if this one dangerous element was now eliminated from the field, and that hereafter storage batteries as a motor power will be relegated to a back seat.

Dr. J. Rollo Knapp, of New Orleans, had a very elaborate and complicated array of electric apparatus for office and laboratory use, and which justly attracted a great deal of attention. We heard some one remark that he would be afraid to have a tooth filled with such a display of electrical appliances surrounding him. It did look more like the office of a conjurer than that of a practical dentist. No one, however, doubts Dr. Knapp's ability to use all the appliances in his possession, and our unappreciation only shows how much we are missing in not introducing more labor saving devices into our daily practice. The days of small things are rapidly passing away, and "hand pressure" and "fine points" are being laid aside or buried with their victims. We speak feelingly of this matter because of our own experience in that misdirected line of manipulation. Any man who will provide himself with either one of Dr. W. G. A. Bonwell's pluggers, and master their use, will add ten effective years to his life.

Dr. C. Edmund Kells, Jr., of New Orleans, had a very compact arrangement of the most essential application of electricity to office use, one new and very convenient appliance being a tiny incandescent light attached to the ordinary mouth mirror for examinations. An automatic reel winds up his cords, when not in use, out of sight, in a neat little cabinet, which also holds all the instruments to which the electric current is applied. Among them is Dr. Whitefield's device for bleaching teeth, by the liberation of chlorine gas from common salt and water in the cavity of the tooth.

The largest exhibitors were the S. S. White Co.; Claudius Ash & Sons; the Keller Medicine Co's. (dental specialties); the Welch Dental Co.; the American Manufacturing Co.; Lambert Pharmaceutical Co. (Listerine); Gideon Sibley; R. S. Williams; H. D. Justi; the Florence Manufacturing Co. (Ideal tooth brushes); Dr. I. W. Ivory (new rubber dam clamp and napkin holder); Durand & Co., with Ward's electro-metallic dental plate, of gold and silver, made by electro deposits directly upon the cast, forming a perfectly fitting plate of silver with pure gold surfaces.

The exhibit of the S. S. W. Dental M'f'g Co. contained How's porcelain inlays, more especially adapted for labial cavities on the incisors; a gold plate-rimmer, a well-known tinsmith's tool adapted to dental use, to do away with soldering the rim on gold plates; two new rubber-dam clamps. How's cervix clamp and Johnson's lever clamp both are designed to hold the gum back from a cavity under the gum border, the latter also holding the lips out of the way. Robinson's collar pliers, designed to facilitate the work of shaping and contouring bands in crown work; Dr. Kirk's sterilizing apparatus for implantation operations, by which the sterilizing baths are automatically and accurately held at the same constant temperature. Their Primrose folding screen is a very practical device; the curtain-screen is hung on cords fixed at one end to the wall, and at the other end to a spring-roller in a an upright hollow shaft, mounted on a base with castors, closing the screen automatically by pressure on a brake-handle. Among their very large assortment of teeth are very thin yet strong teeth ground out on the lingual side ready for use in hurried rubber work, saving much time and labor in grinding; also some extremely artistic hand-made "old-people's teeth," reproducing the characteristic coloring and attrition of old age.

Gideon Sibley showed an entirely new form of gold called Sibley's Felt Gold, a thoroughly cohesive and homogeneous gold, spreading readily and conforming perfectly to the walls of the cavity, making a perfectly tight filling.

Claudius Ash & Sons had on exhibit many new forms of teeth; they also make a specialty of forceps, in great variety of form.

The American Manufacturing Co. had a very large display in new forms of instruments.

The Keller Medicine Co. had on display a great variety of amalgams, cements, dentrifices and many new preparations, including their dental resins, iodized, carbolized, morphiated, salicylated, capsicum plasters, etc., spunk, pledgets, styptic and other cottons. As is well known, all of their preparations are non-secret, having the formula printed on every package. They have also the "Gould" dental chair, the "new improved Gould," offering many superior features in movements and positions. It can be laid down horizontally for the administration of anesthetics, forming a flat table, or in case of chloroform narcosis, it can be tilted backward until the patient's head is two feet lower than the knees, making it unnecessary to remove the patient from the chair in case of emergency. This is the only dental chair which can be adjusted to these positions. It can also be tilted downward and forward.

The advantages of the Pemberthy Bracket will be readily appreciated. Instead of the usual drawers, this bracket has two trays, and four slides containing ten drop bottoms, ranging in depth from short drops for engine burs to a depth sufficient for Varney pluggers, etc. When the slides are drawn out, the points of some 120 instruments are exposed to view, in a perpendicular position, each one in its own compartment.

Their "dry-grinding" corundum wheels and points, for the dental engine or laboratory lathe, will be found very durable, and much superior to the ordinary shellac sheets.

They have the exclusive sale of Wooley's Electro-Magnetic Dental Engine. This engine is very light, starts easily, having no dead centres, can be instantly reversed, or brought to a dead stop in less than two revolutions, while running at 2000 a minute, and is under perfect control of the operator by means of a foot pedal switch-board and brake. It has no rapidly revolving wheel, and makes no buzzing noise to frighten nervous patients.

*Dr. W. H. Richards* (Knoxville, Tenn.) had on exhibition a new abscess syringe, which, in addition to the large receptacle for tepid water, has a smaller one holding a few drops of the remedy to be used. The remedy is carried ahead of the water by pressure on the small bulb, and can be preceded or followed by water, without removal from the mouth, and without any bubbling of air. It has points of different sizes and shapes for treating root canals, fistulous tracts, blind abscesses, antrum, etc.

*Dr. Custer* (Springfield, Ohio) had a new atomizer for ether-spray, etc., by which the flow of ether is controlled at will. The ether is thrown into a glass cylinder holding enough for three operations.

